# The Mechanism of *Mycobacterium smegmatis* PafA Self-Pupylation

**DOI:** 10.1371/journal.pone.0151021

**Published:** 2016-03-08

**Authors:** Xuejie Chen, Chandan Li, Li Wang, Yi Liu, Chuanyou Li, Junjie Zhang

**Affiliations:** 1 The Key Laboratory of Cell Proliferation and Regulation Biology of Ministry of Education, Institute of Cell Biology, College of Life Sciences, Beijing Normal University, Beijing 100875, China; 2 Department of Bacteriology and Immunology, Beijing Tuberculosis and Thoracic Tumor Research Institute/Beijing Chest Hospital, Capital Medical University, Tongzhou District, Beijing, China; The Chinese University of Hong Kong, CHINA

## Abstract

PafA, the prokaryotic ubiquitin-like protein (Pup) ligase, catalyzes the Pup modification of bacterial proteins and targets the substrates for proteasomal degradation. It has been reported that that *M*. *smegmatis* PafA can be poly-pupylated. In this study, the mechanism of PafA self-pupylation is explored. We found that K320 is the major target residue for the pupylation of PafA. During the self-pupylation of PafA, the attachment of the first Pup to PafA is catalyzed by the other PafA molecule through an intermolecular reaction, while the formation of the polymeric Pup chain is carried out in an intramolecular manner through the internal ligase activity of the already pupylated PafA. Among the three lysine residues, K7, K31 and K61, in *M*. *smegmatis* Pup, K7 and K31 are involved in the formation of the poly-Pup chain in PafA poly-pupylation. Poly-pupylation of PafA can be reversibly regulated by depupylase Dop. The polymeric Pup chain formed through K7/K31 linkage is much more sensitive to Dop than the mono-Pup directly attached to PafA. Moreover, self-pupylation of PafA is involved in the regulation of its stability *in vivo* in a proteasome-dependent manner, suggesting that PafA self-pupylation functions as a mechanism in the auto-regulation of the Pup-proteasome system.

## Introduction

In eukaryotic cells, ubiquitin is post-translationally attached to many cellular proteins as a single moiety or in the form of polymeric chains. The attachment of the ubiquitin chain, termed poly-ubiquitination, targets substrate proteins to undergo 26S proteasome-mediated degradation [1, 2]. Ubiquitination is carried out by three enzymes, namely, ubiquitin-activating enzyme (E1), ubiquitin conjugating enzyme (E2), and ubiquitin ligase enzyme (E3). The E3 ligase is responsible for transferring ubiquitin from E2 to target substrates, endowing the ubiquitin-proteasome system (UPS) with its high substrate specificity [3]. A typical feature of most eukaryotic E3 ligases is targeting for degradation via self-catalyzed ubiquitination or through ubiquitin modification mediated by an external ligase, which plays a critical role in the regulation of the ubiquitin system [4]. The eukaryotic UPS is involved in a large variety of cellular activities, with critical functions in the stress response, cell cycle control, protein quality control, signal transduction, the immune response, and many other biological processes.

Proteasomes also exist in archaea and bacteria, especially in the large and diverse group of actinobacteria, including *Mycobacterium* [5, 6]. In mycobacteria, a prokaryotic ubiquitin-like protein (Pup) has been identified, which can modify protein substrates on the lysine residue and direct them towards proteasome-mediated degradation [7, 8]. Even though its function analogous to that of ubiquitin, Pup is an intrinsically disordered small protein and does not exhibit any structure and sequence homology to ubiquitin [9, 10]. Two enzymes are required for the pupylation process. First, the C-terminal glutamine residue of Pup^Q^ is deamidated to glutamate by deaminase of Pup (Dop) to form Pup^E^ [11, 12], and then proteasomal accessory factor A (PafA) is responsible for activating Pup^E^ and conjugating Pup^E^ to substrate protein [13–15]. In a manner analogous to the reversible regulation of ubiquitination by deubiquitinases, Dop functions as a depupylase to remove Pup from pupylated substrates [16, 17]. Pup-tagged protein can be recognized by the N-terminal coiled-coil domain of *Mycobacterium* proteasomal ATPase (Mpa), resulting in unfolding in an ATP-dependent manner and translocation into 20S core proteasomal particle for degradation [18–20]. The entire machinery of Pup-mediated protein proteasomal degradation is named the Pup-proteasome system (PPS). PPS is involved in the survival of *Mycobacterium smegmatis* under starvation [21] and is essential for the full virulence and persistence of *Mycobacterium tuberculosis* in its host [22–26].

Ubiquitination is mediated by hundreds of E3 ubiquitin ligases in eukaryotes; however, pupylation is carried out by a single ligase PafA in prokaryotes [7]. PafA belongs to the carboxylate-amine ligase superfamily [27], consisting of a large N-terminal domain with a central β-sheet as the active site packed against a cluster of helices and a smaller unique C-terminal domain [28]. The crystal structure of *Corynebacterium glutamicum* PafA complexed with Pup^E^ reveals that Pup^E^ binds to the groove wrapped around PafA and positions the C-terminal glutamate in the active site of PafA [29]. PafA catalyzes a two-step reaction by forming a γ-glutamyl phosphate-mixed anhydride intermediate on the C-terminal glutamate of Pup^E^ by hydrolyzing ATP, followed by attaching it to nucleophilic substrates by catalyzing the formation of isopeptide bonds between Pup^E^ C-terminal glutamate γ-carboxylate and the side chain of protein substrate lysine residues [13]. Mass spectrometry has identified 55 and 41 pupylated proteins in *M*. *tuberculosis* and *M*. *smegmatis*, respectively [30, 31], most of which appear to only have one site of lysine modification, with a few exceptions having two or more pupylation sites. There is no conspicuous motif near the modified lysine residue. The way in which PafA recognizes these proteins as substrates for pupylation is still unknown.

As the only Pup ligase, PafA is the regulatory hub that controls the pathway of bacterial PPS [7]. It has been reported that PafA can be poly-pupylated by itself [21] and that PafA accumulates in the strain with *mpa* [26] or *prcBA* [21] deletion. Other PPS components, including Mpa, Dop and the 20S α subunit, also serve as the substrates of PafA [21, 32]. Unlike the eukaryotic UPS, in which poly-ubiquitin chains target proteins to the 26S proteasome, in the prokaryotic PPS, mono-pupylation is almost exclusively identified on the pupylated proteins to direct their degradation by proteasomes, and poly-pupylation rarely occurs in substrates *in vivo* [30, 33]. In contrast to other substrates, in this study, we found that *M*. *smegmatis* PafA undergoes self-catalyzed poly-pupylation through a unique mechanism and that the self-pupylation of PafA is involved in the regulation of its stability *in vivo*.

## Materials and Methods

### Bacterial strains and growth conditions

*Escherichia coli* strains were grown in Luria-Bertani broth at 37°C. *Mycobacterium smegmatis* mc^2^155 strains were cultured in Middlebrook 7H9 broth (Difco) supplemented with 0.2% (v/v) glycerol, 0.05% (v/v) Tween-80, and OADC (0.006% oleic acid, 0.5% bovine serum albumin, 0.2% dextrose, 0.0003% catalase and 0.085% sodium chloride) at 37°C. The final concentration of antibiotics were as follows: 25 μg/mL chloramphenicol, 100 μg/mL ampicillin or 100 μg/mL kanamycin for *E*. *coli*, and 50 μg/mL kanamycin or 50 μg/mL hygromycin for *M*. *smegmatis*.

### Plasmids

For protein expression in *E*. *coli*, the gene encoding *M*. *smegmatis* PafA or Dop with N-terminal His_6_-tag was cloned into plasmid pACYCDuet-1 (Novagen), the gene encoding *M*. *smegmatis* Pup^E^ with N-terminal His_6_-tag was cloned into plasmid pET-28a (Novagen), and the gene encoding *M*. *tuberculosis* PanB with N-terminal His_6_-tag was cloned into plasmid pET-21cc (Novagen). For PafA protein expression in *M*. *smegmatis*, the gene encoding *M*. *smegmatis* PafA with N-terminal His_6_-tag was cloned into plasmid pMV261 [34]. For Pup protein expression in *M*. *smegmatis*, the gene encoding *M*. *smegmatis* Pup together with a 198-bp region upstream from its translation start site was cloned into plasmid pACE [35], creating the plasmid-expressed Pup protein under the control of its endogenous promoter. The mutations of the corresponding genes were constructed by site-directed mutagenesis as described previously [36].

### Expression and purification of proteins

*E*. *coli* BL21 cells carrying the corresponding plasmids were grown at 37°C until OD_600_ reached 0.6, and protein expression was then induced by the addition of IPTG to a final concentration of 0.2–0.5 mM at 23°C for 8 h. Cultures were harvested and lysed by sonication with Buffer P1 (50 mM HEPES, 400 mM NaCl, 10% glycerol, pH 8.0). Proteins were purified by affinity chromatography with Ni-NTA His-bind Resin (Novagen) according the standard procedures and stored at -80°C. Wild type and K320R mutant PafA were further purified by size exclusion chromatography on a Superdex 200 Increase 10/300 GL column (GE Healthcare) in Buffer G (50 mM HEPES, 100 mM KCl, 20 mM MgCl_2_, 10% glycerol, pH 8.0).

For PafA purification in *M*. *smegmati*s strains, plasmids encoding N-His_6_ PafA and Pup were co-transformed. Cultures were harvested at OD_600_ ≈ 2.0, re-suspended and lysed by beating with zirconia silica beads in buffer P2 (50 mM Tris-HCl, 400 mM NaCl, 10% glycerol, pH 8.0). Cellular debris was removed by centrifugation at 13000 rpm for 1 h. The PafA protein in the supernatant was purified by affinity chromatography with Ni-NTA His-Bind Resin.

### *In vitro* pupylation and depupylation

For the *in vitro* pupylation reactions, PafA or its variants were incubated with Pup^E^ in buffer R (50 mM HEPES, 100 mM KCl, 20 mM MgCl_2_, 5 mM beta-mercaptoethanol, 10% glycerol, pH 8.0). Reactions were initiated by the addition of 5 mM ATP and incubated at 23°C for the indicated time. Depupylation reactions were carried out by incubating the pupylated protein (Pup∼PafA) and Dop under the same conditions as described in pupylation reactions.

### Construction of gene knockout strains

*M*. *smegmatis* strains with the deletion of the 20S proteasome *β* subunit or *pafA* gene were constructed by homologous recombination with p1NIL and pGOAL19 plasmids, respectively, according to the protocol described previously [37, 38]. The strain with the 20S proteasome *β* subunit gene deletion was designed as *Δβ M*. *smegmatis*, and the strain with the *pafA* gene deletion as *ΔpafA M*. *smegmatis*.

### Protein degradation analysis

Wild-type or *Δβ M*. *smegmati*s strains with PafA and Pup co-expression were used for PafA degradation analysis. When *M*. *smegmatis* cultures were grown at the logarithmic phase (OD_600_ ≈ 0.6–0.8), tetracycline was added to a final concentration of 25 μg/mL to stop protein synthesis. Cultures were harvested at the indicated time points after tetracycline addition, re-suspended and lysed by beating with zirconia silica beads in buffer M (50 mM Tris-HCl, 150 mM NaCl, 10% glycerol, 0.05% Tween-80, 1 mM EDTA, pH 8.0) supplemented with 1× protease inhibitor Cocktail (Roche). Cellular debris was removed by centrifugation at 13000 rpm for 1 h. PafA protein in the supernatant was detected by western blotting with anti-PafA antibody and quantified via gray scanning using ImageJ software. The same amount of supernatant from each culture lysate was subjected to SDS-PAGE as the loading control.

### Antibodies and immunoblotting

Polyclonal antibodies against Pup and PafA were produced by Beijing B&M Biotech Co., Ltd., and synthetic peptides served as antigens for immunization. The anti-Pup antibody was made with the peptide antigen “MAQEQTRRGG,” consisting of the N-terminal 9 amino acid residues in *M*. *smegmati*s Pup. The anti-PafA antibody was made with the peptide antigen “HIWEGVSSATTRSRPII,” consisting of the 174–190 region in *M*. *smegmati*s PafA. The anti-HA antibody was purchased from Sigma-Aldrich. For immunoblotting analysis, protein samples were loaded onto SDS-PAGE for electrophoretic separation, transferred to PVDF membranes, and then subjected to immuno-detection using standard procedures.

### LC/MS analysis

The Pup∼PafA gel band was excised from the SDS-PAGE gel and digested with trypsin. The resulting peptide mixture was subjected to LC-MS/MS analysis in nano LC-LTQ-Orbitrap XL mass spectrometer (ThermoFinnigan, San Jose, CA). Data were collected in a data-dependent mode using the Top 10 strategy and searched using the Proteome Discoverer software 1.4 (Thermo Fischer Scientific). Peptide spectra were searched against an NCBI-derived *M*. *smegmatis* protein database using the Percolator algorithm. Threshold criteria for protein identification were defined as follows: mass accuracy 10 ppm, fragment ion tolerance 0.6 Da, maximum of two missed cleavage sites, and a false discovery rate (FDR) threshold of 1%. The pupylated modification on Lys residue displayed a +243.086 Da mass shift (GGE) for trypsin digestion.

## Results

### Poly-pupylation of PafA

**Fig 1:**
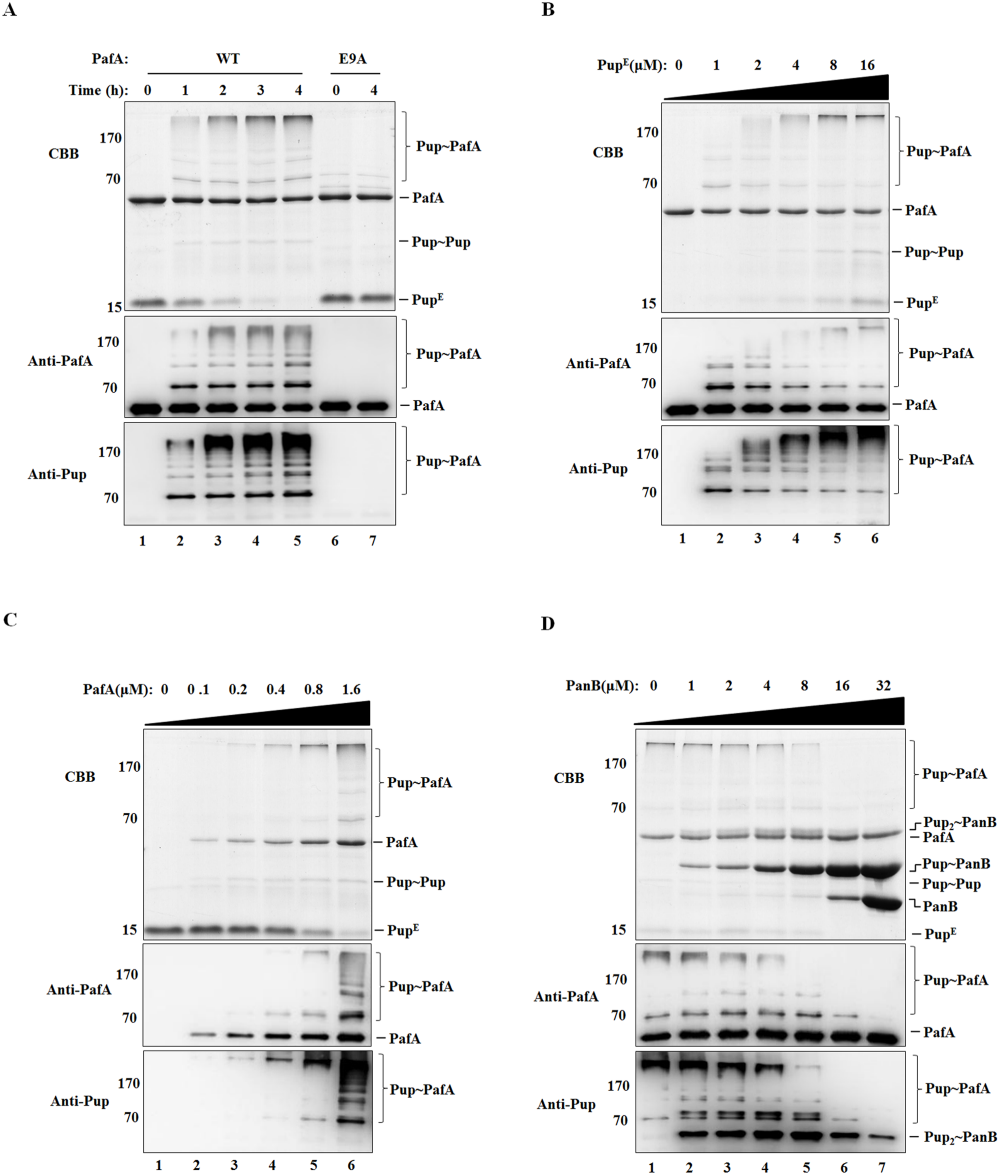
PafA is poly-pupylated *in vitro*. (A) Wild-type PafA or PafA (E9A) (2 μM) were incubated with Pup^E^ (10 μM) in the presence of 5 mM ATP at 23°C for the indicated time points. (B) PafA (1 μM) was incubated with various amounts of Pup^E^ (0 to 16 μM) in the presence of 5 mM ATP at 23°C for 4 h. (C) Various amounts of PafA (0 to 1.6 μM) were incubated with Pup^E^ (10 μM) in the presence of 5 mM ATP at 23°C for 4 h. (D) PafA (1 μM) and Pup^E^ (10 μM) were incubated with various amounts of PanB (0 to 32 μM) in the presence of 5 mM ATP at 23°C for 4 h. Samples were collected and analyzed by SDS-PAGE, followed by Coomassie brilliant blue (CBB) staining or western blotting with anti-PafA and anti-Pup antibody, respectively.

PafA is the only ligase in bacteria to bind Pup^E^ and catalyze protein pupylation. In this study, we tested the possibility of PafA self-pupylation *in vitro* using purified *M*. *smegmatis* PafA and Pup proteins. The Pup protein used has a glutamate at its C-terminus, termed Pup^E^, which can be directly conjugated to the lysine residues in target proteins by PafA. PafA was incubated with Pup^E^ in the presence of ATP, and the reaction products were analyzed by SDS-PAGE and Coomassie Brilliant Blue (CBB) staining. In addition to the bands corresponding to PafA (∼51 kDa) and Pup^E^ (∼15 kDa), distinct bands with high molecular mass were visible at the top of the gel, showing a pattern of poly-pupylation as observed upon poly-ubiquitination. These bands with high molecular mass were detected in the western blot analysis with either anti-PafA or anti-Pup antibody (Fig 1A, lanes 1–5). Mass spectrometry analysis revealed that the resulting high molecular mass band at top of the gel comprised both PafA and Pup (Table 1). The bands with high molecular mass were not produced when the nonfunctional PafA E9A mutant was incubated with Pup^E^ in the presence of ATP (Fig 1A, lanes 6 and 7). These data indicate that PafA can catalyze the poly-pupylation of itself.

**Table 1:** LC-MS/MS analysis of poly-pupylated PafA.

Protein name	Score	% Coverage	PTMs	Modified Lys	Detected pupylated sequences	MH+ [Da]	ΔM [ppm]	XCorr
**PafA (MSMEG_3890)**	422.32	96.68	394					
			6	**K320**	QLDAVESQDFA**k**^**~EGG**^VDTEIDWVIK	2792.36441	0.64	3.45
			10	**K320**	QLDAVESQDFA**k**^**~EGG**^VDTEIDWVIKR	2948.46767	1.33	2.42
			3	**K320**	QLDAVESQDFA**k**^**~EGG**^VDTEIDWVIKRK	3076.56362	1.59	1.57
			5	K162	VLQTP**k**^**~EGG**^AATFCLSQR	1963.00833	0.86	2.57
			1	K162	VLQTP**k**^**~EGG**^AATFCLSQRAEHIWEGVSSATTR	3487.74387	1.45	0.78
			1	K202	DEPHADAE**k**^**~EGG**^YRR	1729.78965	0.42	0.96
			2	K399	A**k**^**~EGG**^LRGEFISAAQEAGR	1947.00565	0.66	2.57
			1	K423	DFTVDWVHL**k**^**~EGG**^LNDQAQR	2328.13767	0.36	1.67
			1	K435	TVLC**k**^**~EGG**^DPFR	1378.67925	0.60	2.43
**Pup (MSMEG_3896)**	213.36	70.24	172					
			3	K7*	GSHMAQEQT**k**^**~EGG**^RGGGGGEDDDLPGASAAGQER	3328.45594	2.35	3.07
			1	K7*	GSHMAQEQT**k**^**~EGG**^R	1515.69841	0.98	2.38
			3	**K31**	E**k**^**~EGG**^LTEETDDLLDEIDDVLEENAEDFVR	3437.56345	0.90	4.51
			3	**K31**	RE**k**^**~EGG**^LTEETDDLLDEIDDVLEENAEDFVR	3593.65903	-0.68	3.57

* *M*. *smegmatis* Pup^E^ protein used for PafA pupylation reaction *in vitro* contains a thrombin cleavage site between N-terminal His_6_-tag and Pup sequence (His_6_-LVPRGSH-Pup). During LC-MS/MS analysis, arginine in the thrombin cleavage site is identified and cleaved by trypsin, leading to the addition of three extra amino acid residues GSH in peptide fragment with Pup K7 modification.

With the increasing concentration of Pup^E^ or PafA, the poly-pupylation of PafA was gradually enhanced in a dose-dependent manner (Fig 1B and 1C). Then, the increasing amounts of the well-known pupylation substrate protein PanB were added together with PafA and Pup^E^ in the presence of ATP. It was found that PafA self-pupylation was suppressed when PanB was added at high concentrations. In addition, unlike the pattern of poly-pupylated PafA, only mono-pupylated PanB and a few di-pupylated PanB (Pup_2_-PanB) can be detected in the reaction system (Fig 1D).

### Mechanism of PafA self-pupylation

**Fig 2:**
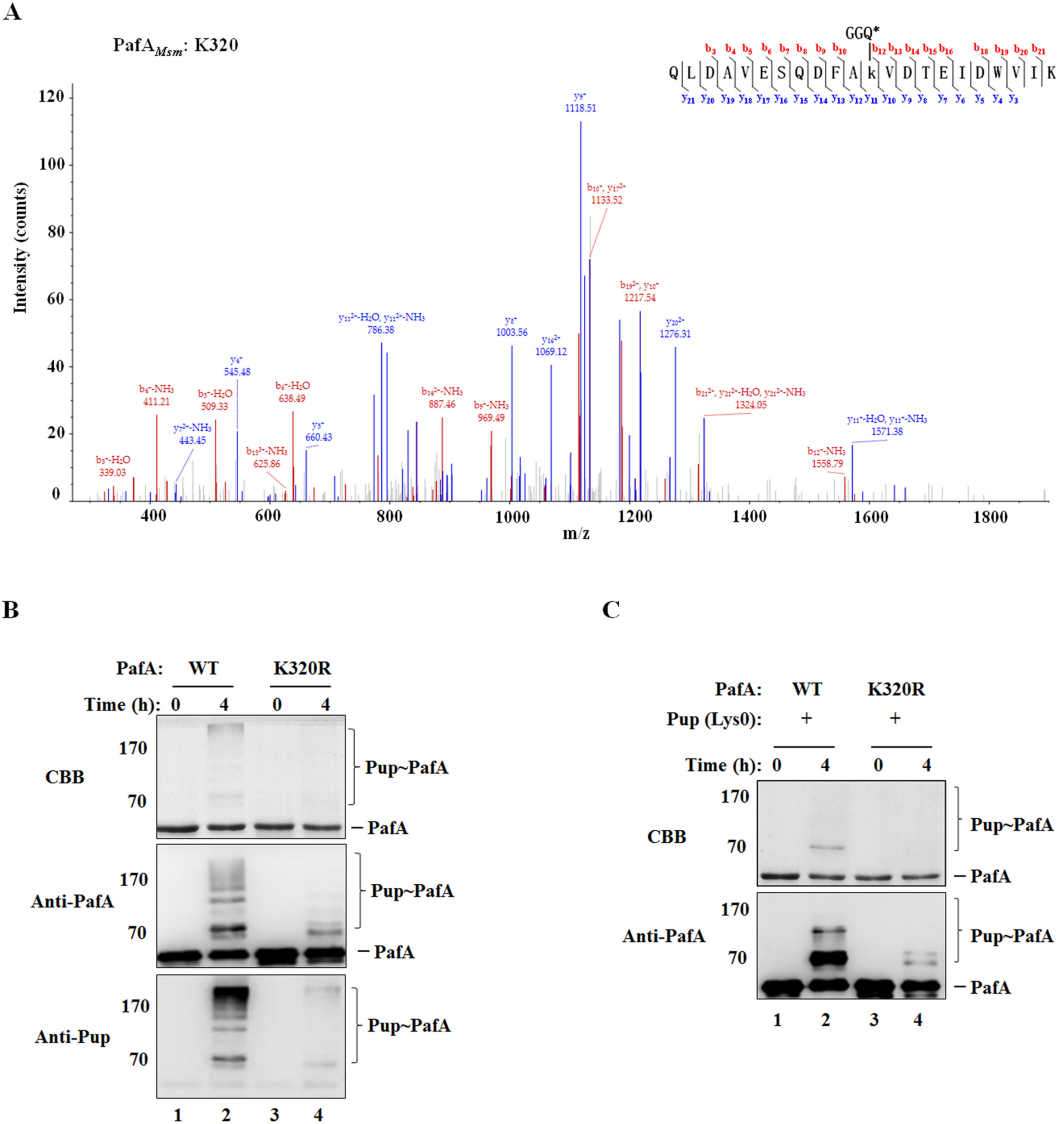
K320 is identified as the major self-pupylation site on PafA. (A) The poly-pupylated PafA band at the top of the gel was excised and subjected to enzyme digestion followed by LC-MS/MS analysis. Pup modification on PafA K320 was detected, displaying a +243.086 Da mass shift (GGE). (B) Wild-type PafA or PafA(K320R) (1 μM) was incubated with Pup^E^ (5 μM) in the presence of 5 mM ATP at 23°C for the indicated times. (C) Wild-type PafA or PafA(K320R) (1 μM) was incubated with Lys0 Pup^E^ (5 μM) in the presence of 5 mM ATP at 23°C for the indicated times. Samples were analyzed by SDS-PAGE, followed by Coomassie brilliant blue (CBB) staining or western blotting with anti-PafA or anti-Pup antibody.

*M*. *smegmatis* PafA has 15 lysine residues. LC-MS/MS analysis revealed that *M*. *smegmatis* PafA can be pupylated at lysine 320 (Fig 2A), as well as at other lysine residues (Table 1). When K320 in PafA was substituted by arginine, both the poly-pupylation of PafA with wild-type Pup^E^ and the mono-pupylation of PafA with Lys0 Pup^E^, in which all of the 3 lysine residues were substituted by arginine, were significantly suppressed (Fig 2B and 2C), indicating that K320 is the main pupylation target in PafA. However, the self-pupylation of PafA K320R mutant was not totally eliminated (Fig 2B, lane 4; Fig 2C, lane 4), suggesting that PafA could be poorly modified by Pup on other lysine residue(s) besides K320 in the *in vitro* reaction system.

**Fig 3:**
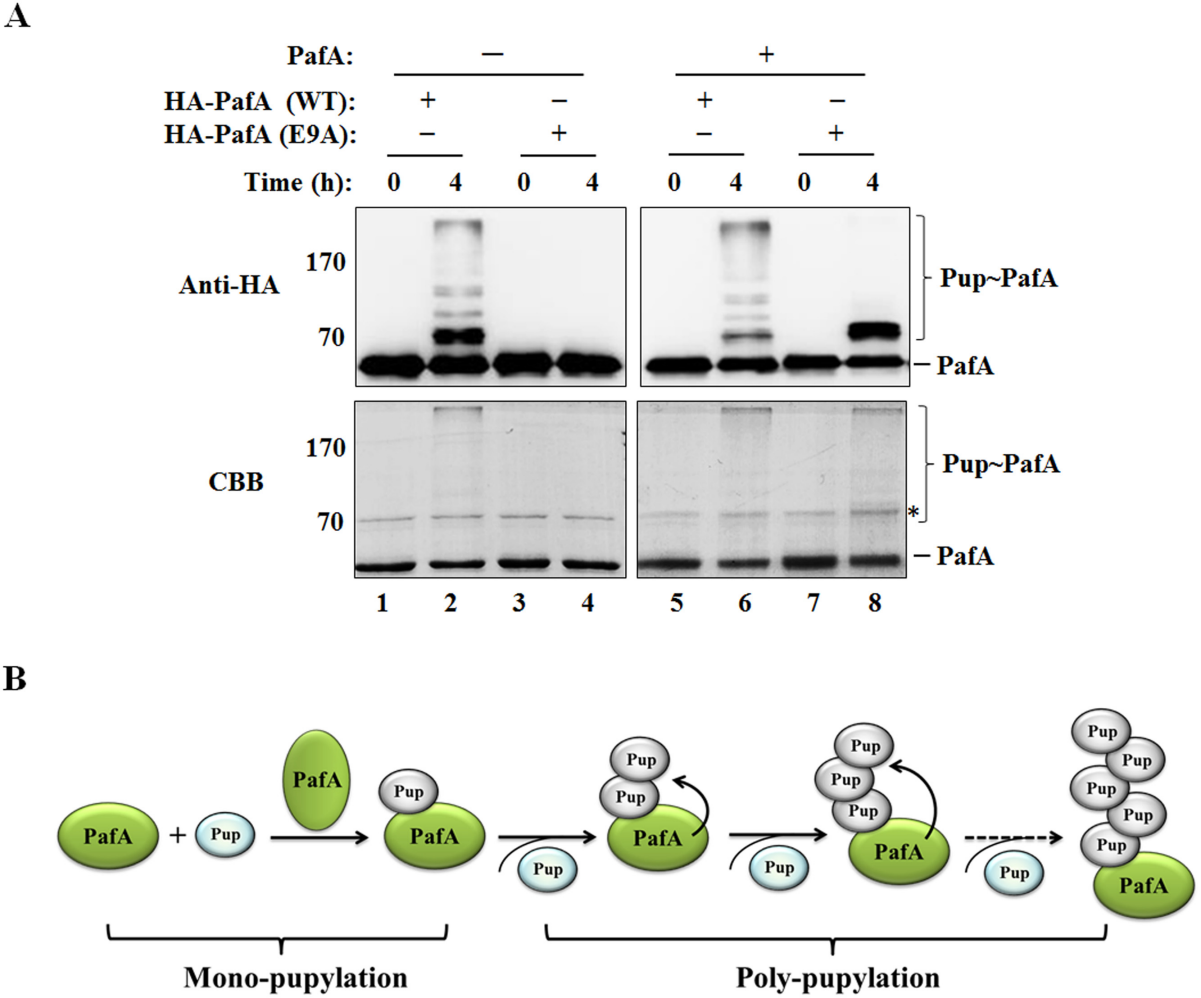
The mechanism of PafA self-pupylation. (A) Wild-type HA-PafA or HA-PafA (E9A) (0.5 μM) was incubated with PafA (0.5 μM) and Pup^E^ (5 μM) in the presence of 5 mM ATP at 23°C for 4 h. Wild-type HA-PafA or HA-PafA (E9A) (1 μM) was incubated with Pup^E^ (5 μM) in the presence of 5 mM ATP as control. Samples were analyzed by SDS-PAGE, followed by Coomassie brilliant blue (CBB) staining or western blotting with anti-HA antibody. (B) Model of the mechanism of PafA self-pupylation. First, PafA is mono-pupylated by the other PafA molecule through an intermolecular reaction, and then the mono-pupylated PafA is further poly-pupylated with its intrinsic ligase activity in an intramolecular manner.

To explore the mechanism of PafA self-pupylation, the wild-type PafA and PafA E9A mutant fused with an N-terminal HA tag were purified. When incubated with Pup^E^ in the presence of ATP, wild-type HA-PafA was poly-pupylated (Fig 3A, lane 2), while the HA-PafA (E9A) mutant without ligase activity, could not be pupylated at all (Fig 3A, lane 4). However, when HA-PafA (E9A) was mixed with wild-type PafA and incubated with Pup^E^ in the presence of ATP, mono-pupylation of HA-PafA (E9A) was observed, but poly-pupylation of HA-PafA (E9A) was not (Fig 3A, lane 8). Similar results were observed when using wild-type PafA and PafA (E9A) fused with the N-terminal Strep tag (data not shown). These results suggest that the mono-pupylation of one PafA can be catalyzed by the other PafA via an intermolecular mechanism, while the formation of a polymeric Pup chain for PafA poly-pupylation cannot, implying that the modification of lysine residues in the PafA-attached Pup is performed in an intramolecular manner depending on the intrinsic ligase activity of the already-pupylated PafA (Fig 3B).

### Pup lysine residues involved in the formation of the Pup chain during PafA poly-pupylation

**Fig 4:**
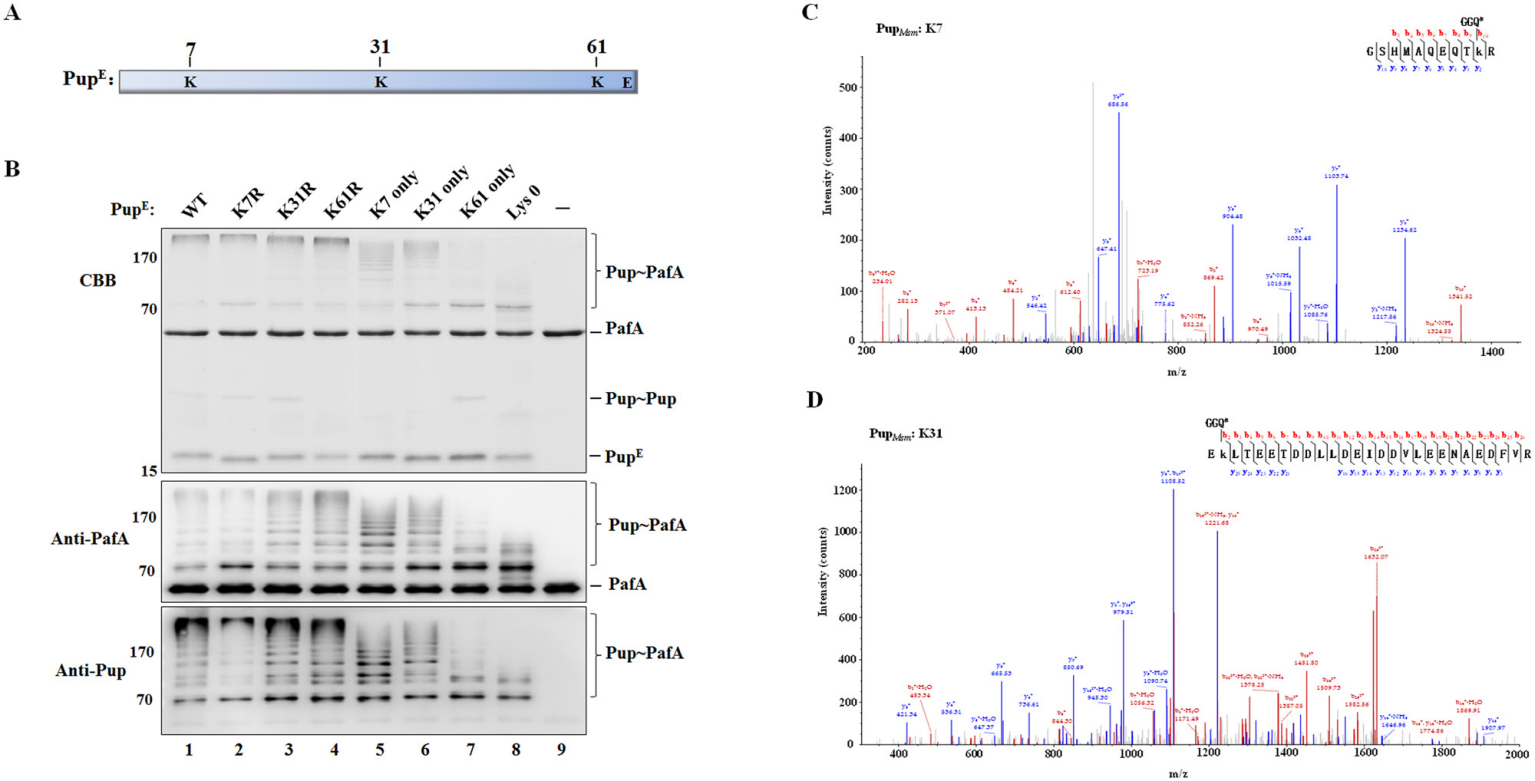
K7 and K31 are the Pup lysine residues involved in the elongation of the Pup chain during PafA poly-pupylation. (A) The positions of lysine residues in *M*. *smegmatis* Pup protein sequences were marked. (B) PafA (1 μM) was incubated with various types of Pup^E^ (5 μM) as indicated in the presence of 5 mM ATP at 23°C for 4 h. Samples were analyzed by SDS-PAGE, followed by Coomassie brilliant blue (CBB) staining or western blotting with anti-PafA and anti-Pup antibody, respectively.

*M*. *smegmatis* Pup contains three lysine residues, K7, K31 and K61 (Fig 4A). A series of Pup^E^ mutants were constructed as indicated in Fig 4B, in which part or all of the lysine residues were substituted with arginine. All of the Pup^E^ mutants have a similar efficiency in conducting the pupylation of PanB (data not shown). To determine the lysine residue(s) of Pup involved in the formation of the Pup chain during PafA poly-pupylation, the Pup^E^ mutants were incubated with PafA in the presence of ATP. PafA poly-pupylation was observed with the use of Pup^E^ mutants containing any two lysine residues (K7R, K31R, and K61R), as well as the Pup^E^ mutant containing K7 or K31 alone (K7 only and K31 only) (Fig 4B, lanes 2–6). The K61 only Pup^E^ performed similarly to the Lys0 Pup^E^ in which all of the lysine residues were substituted with arginines, resulting in the mono-pupylation (or multi-monopupylation) of PafA but not promoting the poly-pupylation of PafA (Fig 4B, lane 7 and 8). These data indicate that K7 and K31 linkages, but not K61 linkages, are involved in polymeric Pup chain formation in PafA poly-pupylation. LC-MS/MS analysis also revealed that K7 and K31 of Pup could be modified as the pupylated sites in poly-pupylated PafA (Table 1, Fig 4C and 4D). However, the existence of K61 may be necessary for the efficiency of PafA poly-pupylation, as the additional K61R mutation in the Pup K7R or K31R mutant further attenuated the poly-pupylation of PafA (Fig 4B, compare lanes 2 and 3 with lanes 5 and 6).

It has recently been reported that Pup can serve as a substrate protein for pupylation [33]. A band corresponding to di-Pup (Pup∼Pup) is visible in Fig 1A–1D and Fig 4B. It is interesting to note that the di-Pup band disappeared whenever a Pup K61 residue was substituted with arginine (Fig 4B, lanes 4, 5, 6 and 8), which is consistent with the results of a previous study demonstrating that free Pup can be pupylated on lysine 61 [33]. Because the poly-pupylation of PafA can be performed with a Pup K61R mutant (Fig 4B, lane 4), the poly-pupylation of PafA is not dependent on the formation of di-Pup.

### Self-pupylation of PafA is reversely regulated by Dop

**Fig 5:**
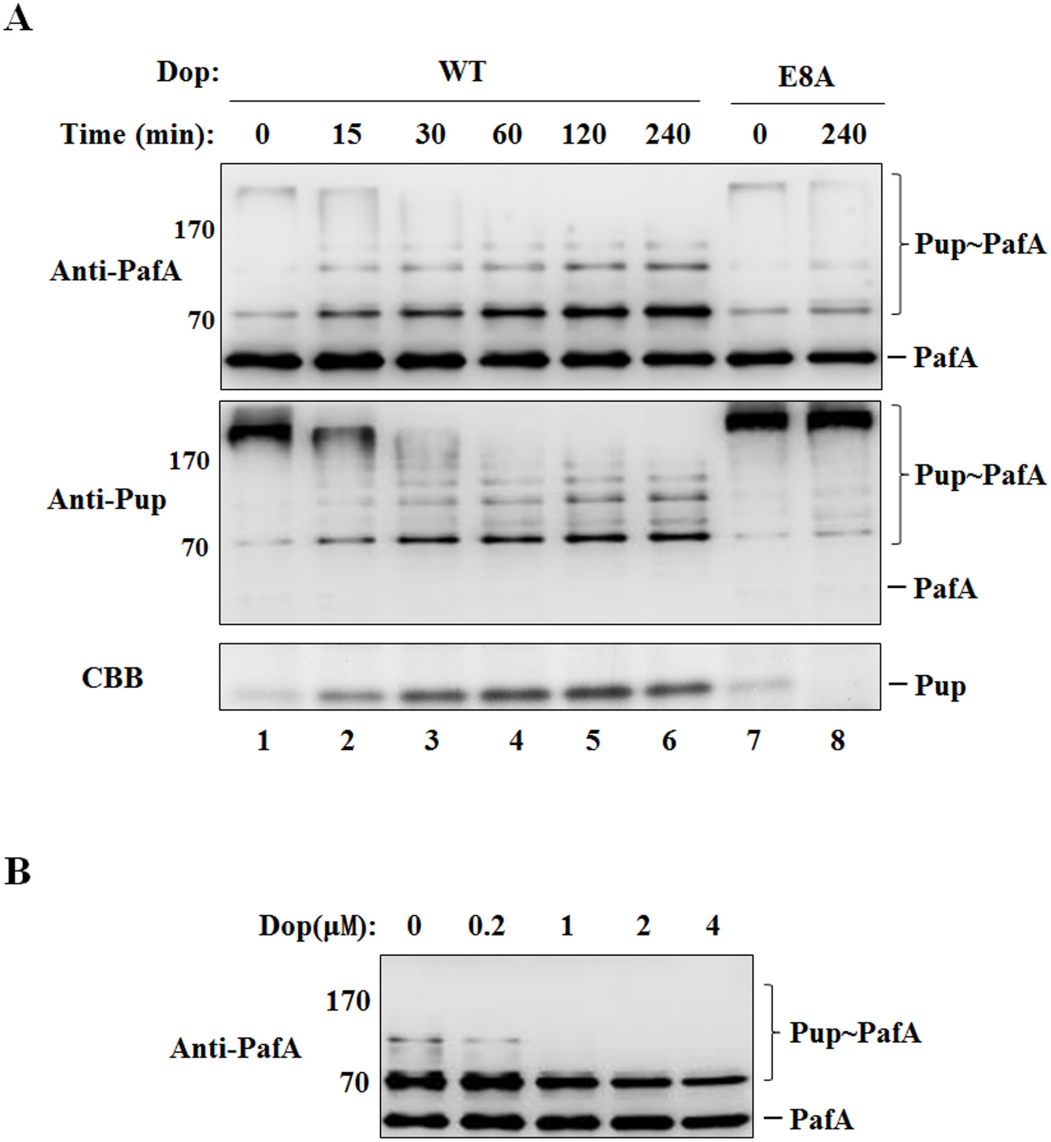
Self-pupylation of PafA is reversibly regulated by Dop. (A) PafA (1 μM) was incubated with Pup^E^ (5 μM) in the presence of 5 mM ATP for 6 h, and then wild-type Dop or Dop (E8A) (1 μM) was added into the reaction mixture, followed by further incubation at 23°C for the indicated times. Samples were analyzed by SDS-PAGE, followed by Coomassie brilliant blue (CBB) staining or western blotting with anti-PafA or anti-Pup antibody. (B) Mono-pupylated PafA was generated by incubating PafA (0.5 μM) and Lys0 Pup^E^ (2.5 μM) in the presence of 5 mM ATP for 6 h, and then the various amounts of Dop (0 to 4 μM) were added into the reaction mixture, respectively, followed by further incubation at 23°C for 4 h. Samples were analyzed by SDS-PAGE, followed by western blotting with anti-PafA antibody.

Dop is a dual-functional enzyme, functioning as deaminase to convert Pup^Q^ to Pup^E^ and also as depupylase to remove Pup from the pupylated proteins. Here, we tested whether the self-pupylation of PafA can be reversely regulated by Dop. First, the self-pupylation of PafA was performed by incubating PafA and Pup^E^ in the presence of ATP for 6 h, and then Dop was added into the reaction mixture. Samples were collected at the indicated time points after Dop addition and analyzed by SDS-PAGE and western blotting with anti-PafA antibody and anti-Pup antibody, respectively. It was found that the polymeric Pup chain in poly-pupylated PafA was disassembled by the depupylase Dop, leading to the accumulation of mono-pupylated PafA (Fig 5A, lanes 1–6). The nonfunctional Dop (E8A) mutant did not have depupylation activity on poly-pupylated PafA (Fig 5A, lanes 7 and 8). To further explore the effects of Dop on mono-pupylated PafA, mono-pupylation (or multi-monopupylation) of PafA was performed by incubating PafA and Lys0 Pup^E^ in the presence of ATP for 6 h, and then increasing amounts of Dop were added into the reaction mixture. Samples were collected at 4 h after Dop addition and analyzed by western blotting with anti-PafA antibody. It was found that mono-pupylated PafA could be depupylated by Dop, but with a low efficiency (Fig 5B). These results indicate that the polymeric Pup chain formed through K7/K31 linkage in the poly-pupylated of PafA is much more sensitive to the depupylase Dop than the mono-Pup directly attached to PafA.

### Self-pupylation of PafA regulates its stability in *M*. *smegmatis*

**Fig 6:**
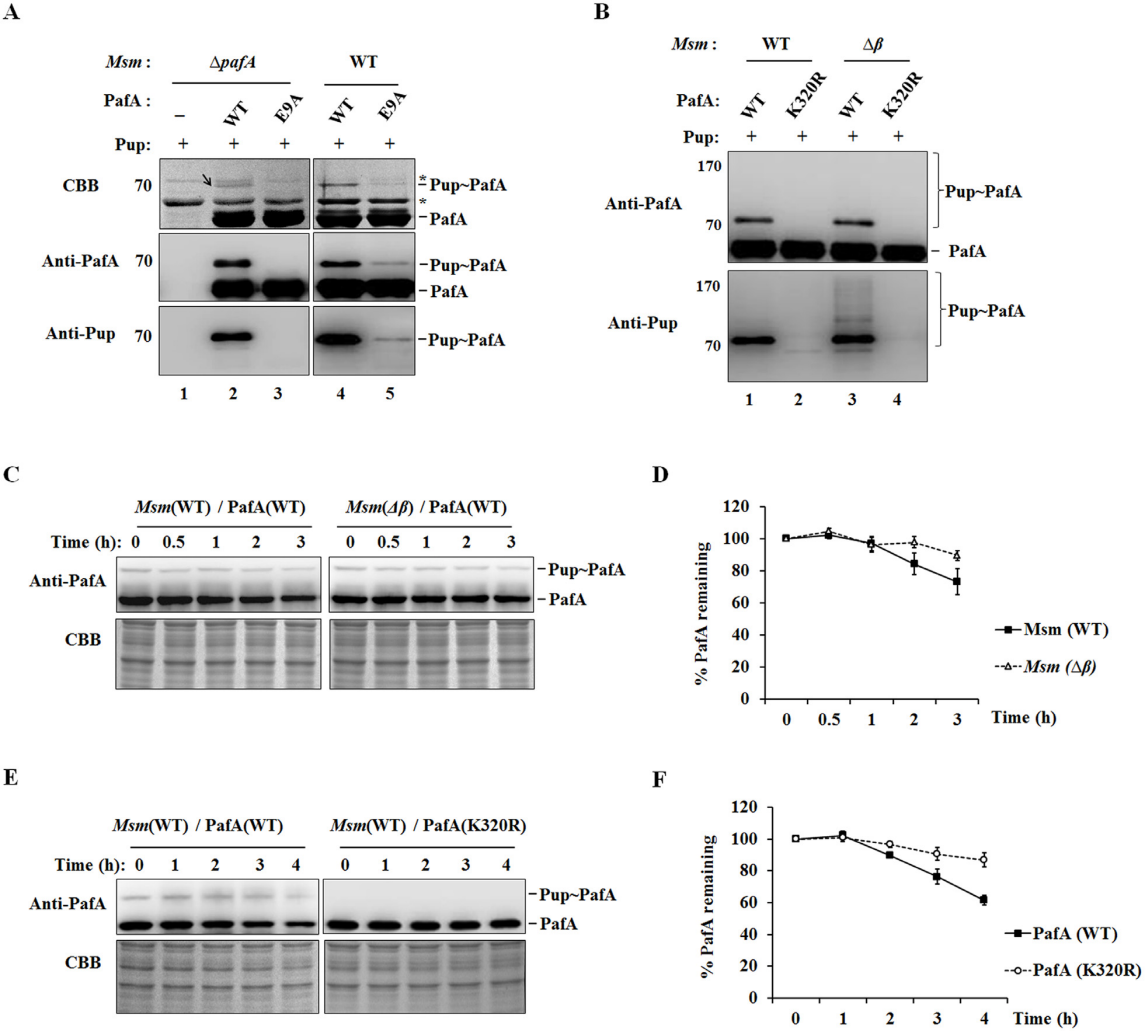
Self-pupylation of PafA regulates its stability in *M*. *smegmatis*. (A) His_6_-PafA or His_6_-PafA (E9A) was co-expressed with Pup in *ΔPafA* or wild-type *M*. *smegmatis*, respectively. His-tagged PafA or PafA (E9A) was purified by Ni-NTA chromatography and analyzed by SDS-PAGE, followed by Coomassie brilliant blue (CBB) staining or western blotting with anti-PafA and anti-Pup antibody, respectively. *ΔPafA M*. *smegmatis* with exogenous Pup but not PafA expression was used as control. Pup∼PafA is indicated by the arrow. The asterisk indicates the non-specific protein band. (B) His_6_-PafA or His_6_-PafA (K320R) was co-expressed with Pup in wild-type or *Δβ M*. *smegmatis*, respectively. His-tagged PafA or PafA (K320R) was purified and detected as described *in* A. (C) His_6_-PafA was co-expressed with Pup in wild-type or *Δβ M*. *smegmatis*. Tetracycline was added into the cultures in the logarithmic phase (OD_600_ ≈ 0.6–0.8) at a final concentration of 25 μg/mL to stop protein synthesis. Cells were collected at the indicated time points after tetracycline addition and lysed. PafA proteins in the supernatants were detected by western blotting with anti-PafA antibody. The same amount of each lysate was subjected to SDS-PAGE as the loading control. Data shown are representative of three independent experiments. (D) Quantitative analysis of the PafA protein detected in C. Error bars were determined by three experimental repeats. (E) His_6_-PafA or His_6_-PafA (K320R) was co-expressed with Pup in wild-type *M*. *smegmatis*, The stability of PafA was detected as described in C. (F) Quantitative analysis of the PafA protein detected in E. Error bars were determined by three experimental repeats.

PafA, the only Pup ligase in Pup-proteasome system, not only catalyzes the Pup modification of substrates but also catalyzes the pupylation of itself. In further studies, the self-pupylation of PafA was identified *in vivo* and its role in the regulation of PafA stability was tested in *M*. *smegmatis*, which contains the whole Pup-proteasome system. The *pafA*-deleted *M*. *smegmatis* was constructed as described in Materials and Methods, termed *ΔpafA M*. *smegmatis*. To identify the self-pupylation of PafA *in vivo*, *M*. *smegmatis* PafA with an N-terminal 6×His tag (His_6_-PafA) was co-expressed with *M*. *smegmatis* Pup in *ΔpafA M*. *smegmatis*, and then total PafA was pulled down with Ni-NTA His-Bind Resin. The mono-pupylation, but not the poly-pupylation, of His_6_-PafA was detected by western blotting with anti-PafA and anti-Pup antibody, respectively (Fig 6A, lane 2). When co-expressed with Pup, the PafA (E9A) mutant without ligase activity could not be pupylated in *ΔpafA M*. *smegmatis* (Fig 6A, lane 3), while the mono-pupylation of PafA (E9A) could occur weakly in wild-type *M*. *smegmatis* (Fig 6A, lane 5), which may be due to the intermolecular effects from endogenous PafA.

With the co-expression of His_6_-PafA and Pup, the poly-pupylation of His_6_-PafA was rarely detected in wild-type *M*. *smegmatis*, but could be observed in the *M*. *smegmatis* with the deletion of the proteasome *β* subunit (*Δβ M*. *smegmatis*) with anti-Pup antibody (Fig 6B, lanes 1 and 3). The poly-pupylation of PafA was not be detected by the anti-PafA antibody, which may be due to the low level of PafA poly-pupylation *in vivo* as well as the less sensitivity of anti-PafA in the detection of poly-pupylated PafA, compared to that of anti-Pup. The protein band of Pup∼PafA was obtained by SDS-PAGE (labeled by the arrow in Fig 6A, upper panel, lane 2) and subjected to mass spectrometry analysis to detect the Pup modification site in PafA *in vivo*. K320 was detected as the only target residue for PafA pupylation *in vivo*. The PafA (K320R) mutant could not be pupylated when co-expressed with Pup in either wild-type or *Δβ M*. *smegmatis* (Fig 6B, lanes 2 and 4).

The stability of PafA was further tested in wild-type and *Δβ M*. *smegmatis*, respectively. It was found that the stability of PafA was increased in *Δβ M*. *smegmatis* compared to that in wild-type *M*. *smegmatis* (Fig 6C and 6D), which is consistent with the previous report [21]. In addition, when expressed in the wild-type *M*. *smegmatis*, wild-type PafA was less stable than PafA (K320R), which could not be pupylated *in vivo* (Fig 6E and 6F). These data suggest that the PafA can be pupylated *in vivo* and that the self-pupylation of PafA is involved in the regulation of its stability.

## Discussion

Pup modification on lysine residue(s) in target proteins, termed pupylation, has been identified as an important way to direct proteins to proteasome-mediated degradation in bacteria belonging to the phyla Actinobacteria and Nitrospira [27]. PafA is the only known ligase to catalyze the pupylation of substrate proteins. To date, hundreds bacterial proteins have been identified to be substrates of pupylation. Different from the eukaryotic ubiquitin-proteasome system, in which poly-ubiquitin chains target proteins to the 26S proteasome, mono-pupylation is almost exclusively identified on the pupylated proteins [30, 33]. The low affinity of PafA to the already pupylated substrate is regarded as the reason for the predominance of mono- over poly-pupylation [33]. However, PafA can catalyze the significant poly-pupylation of itself [21].

In this study, we demonstrate that *M*. *smegmatis* PafA undergoes self-catalyzed poly-pupylation through a unique mechanism, which distinguishes PafA from other substrates. The PafA K320 residue was identified as the main pupylation site *in vitro* and the only pupylation site *in vivo*. The crystal structure of *C*. *glutamicum* PafA has been resolved [28, 29]. According to the high homology between *C*. *glutamicum* PafA and *M*. *smegmatis* PafA, K320 in *M*. *smegmatis* PafA is located away from the ligase activity center, implying that it is impossible for a *M*. *smegmatis* PafA molecule to catalyze Pup ligation on K320 via its own ligase activity. Based on our data in this study, a two-phase mechanism is proposed for PafA self-pupylation. First, the mono-pupylation of PafA is catalyzed by the other PafA ligase in an intermolecular manner, and then the Pup molecule conjugated to PafA is subjected to pupylation to form a polymeric Pup chain in an intramolecular manner through the intrinsic ligase activity of already pupylated PafA. It has been reported recently that IdeR and PanB can be poly-pupylated *in vitro*, but with a much lower efficiency compared to PafA poly-pupylation, which is due to the low affinity of PafA to the already pupylated substrate [33]. During the poly-pupylation of PafA, the interaction between PafA and the already pupylated PafA is not required, leading to the high efficiency of PafA poly-pupylation.

*M*. *smegmatis* Pup have three lysine residues, K7, K31 and K61 (Fig 4A), among which K61 is highly conserved in the C-terminal QKGGQ/E motif of bacterial Pup proteins (S1 Fig). In this study, we found that the K7 and K31 linkages, but not the K61 linkage, are involved in Pup chain elongation during PafA poly-pupylation. K7 and K31 are conserved in the Pup proteins from a group of bacteria, including *M*. *tuberculosis* and *M*. *smegmatis*, most of which contain the entire Pup-proteasome system. In other Pup proteins, except for the lysine residue located in the C-terminal QKGGQ/E motif, there is only one lysine residue in the N-terminal/middle region, or there are no other lysine residues at all. Most bacteria with such types of Pup do not contain proteasomes (S1 Fig). In contrast to PafA poly-pupylation, Pup K61 is the primary target for the poly-pupylation of IdeR and the formation of di-Pup from free Pup [33]. During poly-ubiquitination in eukaryotic cells, ubiquitin chains are formed with distinct conformations with specific linkages between ubiquitin molecules, acting as a code to determine the substrate’s fate in the cells [1]. The different linkages in the polymeric Pup chain during poly-pupylation may have a biological role, which remains to be elucidated.

The E3 ubiquitin ligase in eukaryotes can be targeted for degradation by self-ubiquitination or through ubiquitin modification mediated by an external ligase, which plays a critical role in the regulation of the ubiquitin system [4]. Based on our results and the previous report [21], self-pupylation of PafA is also involved in its stability regulation in a proteasome-dependent manner *in vivo*. However, to date, we have not clearly distinguished the contribution of mono-pupylation and poly-pupylation to PafA degradation. On the other hand, PafA self-pupylation is suppressed in the presence of the substrate protein PanB at high concentrations (Fig 1D) and disassembled by the depupylase Dop (Fig 5A and 5B). Compared to the *in vitro* results, the poly-pupylation of PafA *in vivo* is much weaker in *Δβ M*. *smegmatis* (Fig 6B, lanes 1 and 3), which also may be due to the suppression effects of abundant substrate proteins, as well as the presence of Dop. We propose that the self-pupylation of PafA may be promoted when substrate or Dop protein levels are decreased and functions as a negative feedback loop to prevent the uncontrolled tagging and degradation of cellular proteins. Therefore, the self-pupylation of PafA functions as an important mechanism in the auto-regulation of the Pup-proteasome system.

## Supporting Information

S1 Fig Sequence alignments of Pup proteins. The amino acid sequences of Pup proteins were downloaded from the NCBI GeneBank and aligned with DNAMAN software. Lysine residues in Pup protein sequences were indicated by squares.
(TIF)
