# Supplementary figures and images for: The Mechanism of Mycobacterium smegmatis PafA Self-Pupylation
Source: PLoS One. 2016 Mar 8;11(3):e0151021. doi: 10.1371/journal.pone.0151021 (PMC4783102; doi:10.1371/journal.pone.0151021)

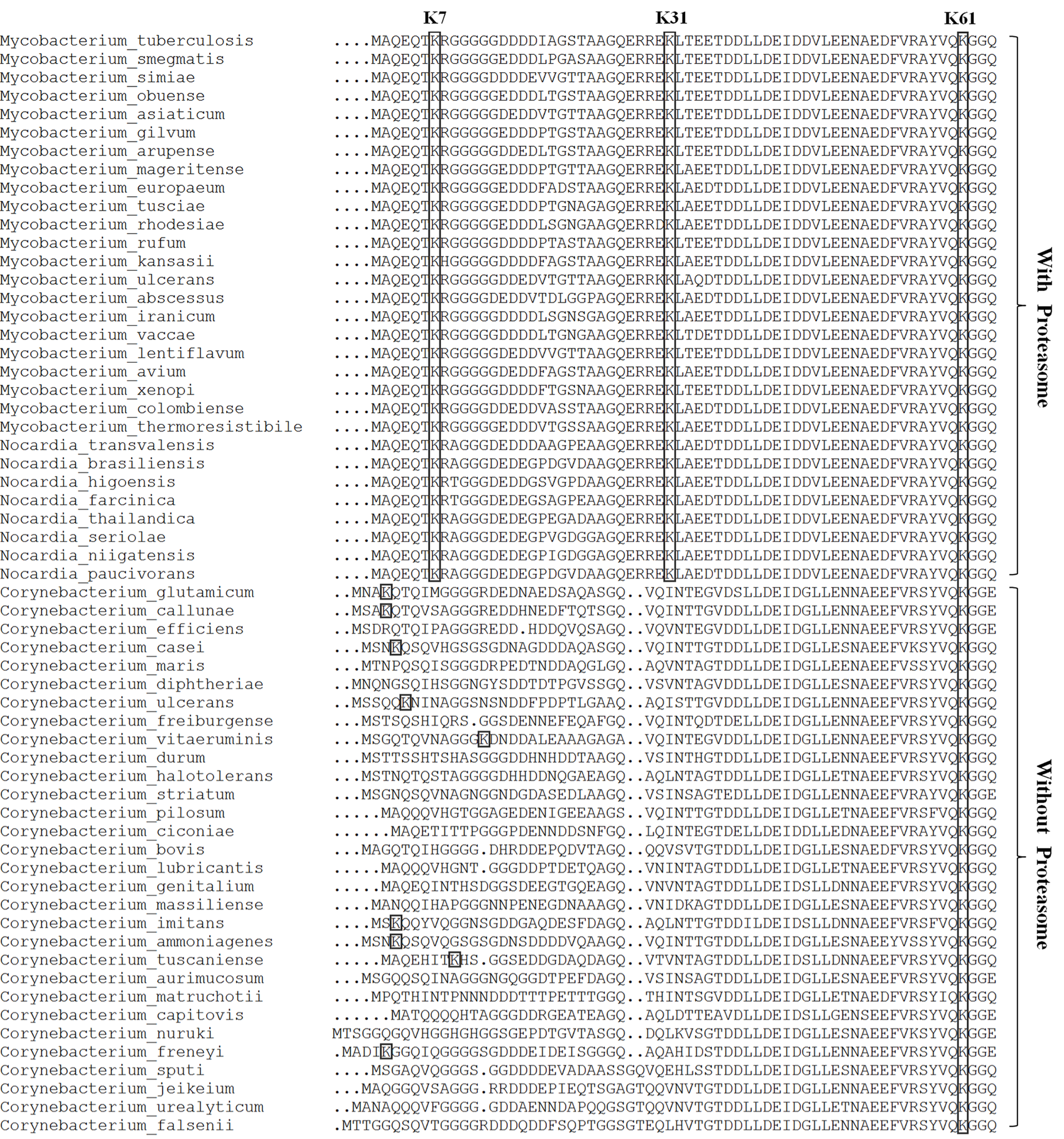

Supplement: S1 Fig — The amino acid sequences of Pup proteins were downloaded from the NCBI GeneBank and aligned with DNAMAN software. Lysine residues in Pup protein sequences were indicated by squares. (TIF) [file pone.0151021.s001.tif]
